# Physical characterization and antioxidant activity of thymol solubilized Tween 80 micelles

**DOI:** 10.1038/srep38160

**Published:** 2016-12-01

**Authors:** Ling-Li Deng, Maierhaba Taxipalati, Fei Que, Hui Zhang

**Affiliations:** 1 College of Biosystems Engineering and Food Science, Fuli Institute of Food Science, Zhejiang Key Laboratory for Agro-Food Processing, Zhejiang R&D Center for Food Technology and Equipment, Zhejiang University, Hangzhou 310058, China; 2 Turpan Vocational and Technical College, Turpan 838000, China; 3 Department of Applied Engineering, Zhejiang Economic and Trade Polytechinc, Hangzhou 310018, China

## Abstract

Attempts were made to solubilize thymol in Tween 80 micelle to study the solubilization mechanism of thymol and the effect of solubilization on its antioxidant activity. The maximum solubilized concentration of thymol in a 2.0% (w/v) Tween 80 micelle solution is 0.2 wt%. There was no significant difference in Z-average diameter between the empty micelles and thymol solubilized micelles. ^1^H NMR spectra indicated that 3-H and 4-H on the benzene ring of thymol interacted with the ester group between the hydrophilic head group and the hydrophobic tail group of Tween 80 by Van der Waals’ force. Ferric reducing antioxidant potential (FRAP) and cupric ion reducing antioxidant capacity (CUPRAC) assays showed that the reducing antioxidant activity of free thymol did not change after solubilized in Tween 80 micelles. Compared to free thymol, the solubilized thymol showed higher activities to scavenge DPPH (2,2-diphenyl-1-picrylhydrazyl) and hydroxyl radicals. The present study suggested a possible preparation of thymol-carrying micelles with enhanced antioxidant activities that could be applied in food beverages.

Thymol, a monoterpene phenol generally isolated from *Thymus vulgaris L.* and *Origanum vulgare L.*, has attracted researchers’ attention for various biological activities, including the bactericidal, fungicidal, and insecticidal efficacies or its ability to enhance biological efficacy of other monoterpenes^1^. It possesses excellent antioxidant properties due to the presence of phenolic hydroxyl group in its structure, which is known to exhibit potent antioxidant activity by absorbing and neutralizing free radicals^2^,^3^. As thymol is a generally regarded as safe (GRAS) food additive used in USA, Europe and China, many studies have been conducted to show that thymol is a potential alternative to synthetic antioxidants in the food matrix. Hossain *et al*.^4^ compared the antioxidant activities of 30 spice extracts to 5 popular synthetic antioxidants and found that thymol was comparable to most of the synthetic antioxidants. Quiroga *et al*.^5^ reported that thymol provided antioxidant protection to roasted sunflower seeds and showed a comparative antioxidant effect to butylated hydroxytoluene (BHT). An *in vivo* study has found that thymol-fed rats maintained significantly higher antioxidant enzyme activities and total antioxidant status, which highlighted the potential benefits of thymol as a dietary antioxidant^6^. Giannenas *et al*.^7^ reported that the dietary thymol supplementation in rainbow trout significantly reduced NO serum levels and increased antioxidant protective capacities in the trout fillets during 5 days of refrigerated storage.

In spite of the effective antioxidant activity at low concentrations *in vivo* and *in vitro*, thymol still sees a limited application in food industry due to its hydrophobicity that makes it difficult to uniformly disperse in food matrices. On the other hand, thymol is used as one of aromatic substances to enrich foods with typical aromas and tastes, these characteristics may prevent its further applications in food industry. Solubilization of thymol in surfactant micelles may be a possible way to solve the problem. Surfactants dispersed in aqueous solutions, above the critical micelle concentration, can spontaneously form micelles, which have been extensively utilized for solubilization of hydrophobic bioactives and nutrients. Other than improving solubility, the encapsulation of thymol in surfactant micelles have been studied for controlled release of volatiles^8^,^9^. Solubilization of antioxidants in different phases and environments of micelles may result in different physicochemical interactions compared to homogeneous systems thereby strongly influencing their activity^10^,^11^,^12^. Heins *et al*.^13^ reported that the antioxidant activity of phenolic compounds depended upon their interaction site at the interface of micelles. The dispersion of radical or antioxidant within micelles might serve as a physical barrier to the antioxidant reaction. Guo and Wei^14^ found that the antioxidant parts in the rutin molecule were shielded by cetyltrimethyl ammonium bromide (CTAB) micelles, leading to a decrease in hydroxyl radical scavenging activity. Chat *et al*.^15^ reported that rutin in cationic (CTAB, TTAB, DTAB), non-ionic (Brij 78, Brij 58, Brij 35) and anionic (sodium dodecyl sulfate) micelles had a lower DPPH radical scavenging activity (RSA) compared to that in methanol, and it also exhibited an enhanced DPPH RSA in mixed cationic-non-ionic micelles compared with any of the single micelles. Miguel *et al*.^16^ evaluated the antioxidant activities of thymol and the corresponding β-cyclodextrin complexes, and found that thymol β-cyclodextrin complexes maintained its capacity for scavenging the free radical using ORAC method, but the β-cyclodextrin complexes had only 40% of trolox equivalent antioxidant capacity compared to the origin.

The objective of this study was to characterize the solubilization of thymol in non-ionic Tween 80 micelles and investigate the solubilization effect on the antioxidant activity of thymol. ^1^H NMR was conducted to study the location of thymol in Tween 80 micelles. The antioxidant activities of the free and solubilized thymol were studied by ferric reducing antioxidant potential (FRAP), cupric ion reducing antioxidant capacity (CUPRAC), DPPH radical scavenging activity, and hydroxyl radical scavenging activity.

## Results and Discussion

### Maximum additive concentration of thymol

**Figure 1:**
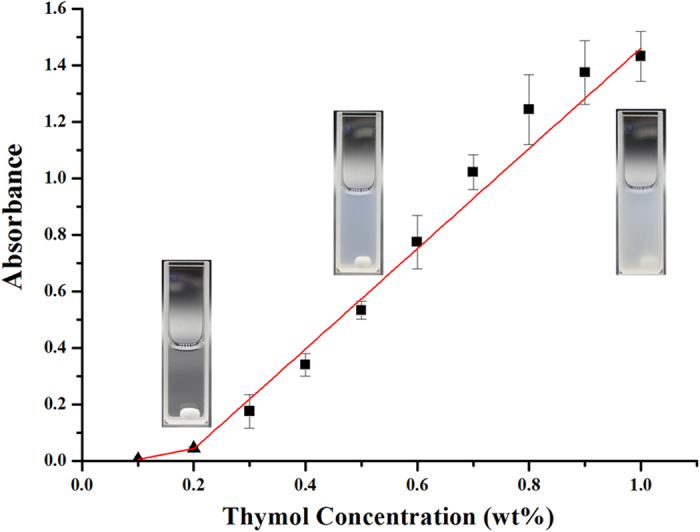
Turbidity of the solutions prepared with various concentrations of thymol in 2.0% (w/v) Tween 80 solution.

The maximum additive concentration (MAC) was defined as the highest concentration of a lipophilic compound that could be incorporated into a micellar surfactant solution at a given surfactant concentration^17^. The absorbance of 2.0% (w/v) Tween 80 micelle solution with the titration of thymol was shown in Fig. 1. The absorbance remained almost zero as thymol was titrated into the solution until a critical concentration was reached. The absorbance then increased abruptly, indicating that micelles were fully saturated and no longer able to take up more thymol. The increased absorbance indicated the formation of droplets of insolubilized thymol that was dispersed in the aqueous phase. The concentration at which the turbidity increased was defined as MAC. The maximum amount of thymol that could be incorporated into 2.0% Tween 80 micelles is 0.2 wt%. The thermodynamic driving force for the solubilization is the reduction of the free energy of the system through the optimization of curvature of the micelle and the decrease in contact area between the non-polar solubilizate and the polar solvent^18^.

Encapsulation of thymol in Tween 80 micelles may not only facilitate the solubilization of thymol in polar solvents, but also decrease the mouth irritation by blocking its intimate contact with mouth tissues. In spite of the fact that thymol is a GRAS approved food additive, some researches indicated that thymol could cause irritation of mouth tissues because of its membrane affinity and hydrophobicity^19^,^20^. It has been reported recently that micelle encapsulation could alleviate irritation of bioactive compounds, such as capsaicin^21^ and menthe oil^22^. Although the side effects of thymol might be alleviated by encapsulation, the safety should be still thoroughly assessed before the practical applications in food systems.

### Particle size distribution

**Figure 2:**
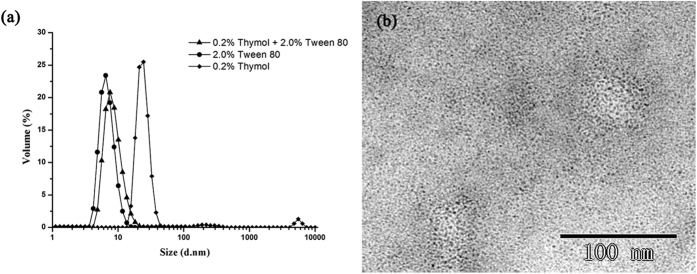
(**a**) Droplet size distribution of 0.2% thymol in water (♦), 2.0% Tween 80 solution (●), and 0.2% thymol solubilized in 2.0% Tween 80 solution (▲), (**b**) TEM image of Tween 80 micelles fully solubilized with thymol.

The particle size distributions of 2.0% Tween 80 micelles, 0.2% thymol solubilized Tween 80 micelles, and 0.2% thymol in water were shown in Fig. 2a. The empty and solubilized micelles showed a similar monomodal distribution, with the Z-average diameter of about 10 nm and polydispersity index of around 0.26. As evidenced by the morphology observation of the solubilized micelles by transmission electron microscopy in Fig. 2b, it could be clearly seen that the Tween 80 micelles fully solubilized with thymol were well-distributed with spherical nanostructures. In comparison, the particle size distribution of thymol in water is not monomodal, and thymol aggregates might attribute to the small peak around 5000 nm. The Z-average diameter and polydispersity index of thymol in water were 178 nm and 0.63, indicating that the free thymol molecules were not uniformly dispersed in water. The addition of thymol to Tween 80 micelles led to a slight increase in the average diameter, compared to the empty micelles. The diameter increase after encapsulating molecules within micelles has also been observed in many other studies^18^,^23^,^24^. This observation indicated that thymol has been successfully incorporated in Tween 80 micelles, which makes the micelle structure more stretched.

### The location of thymol in Tween 80 micelles

**Figure 3:**
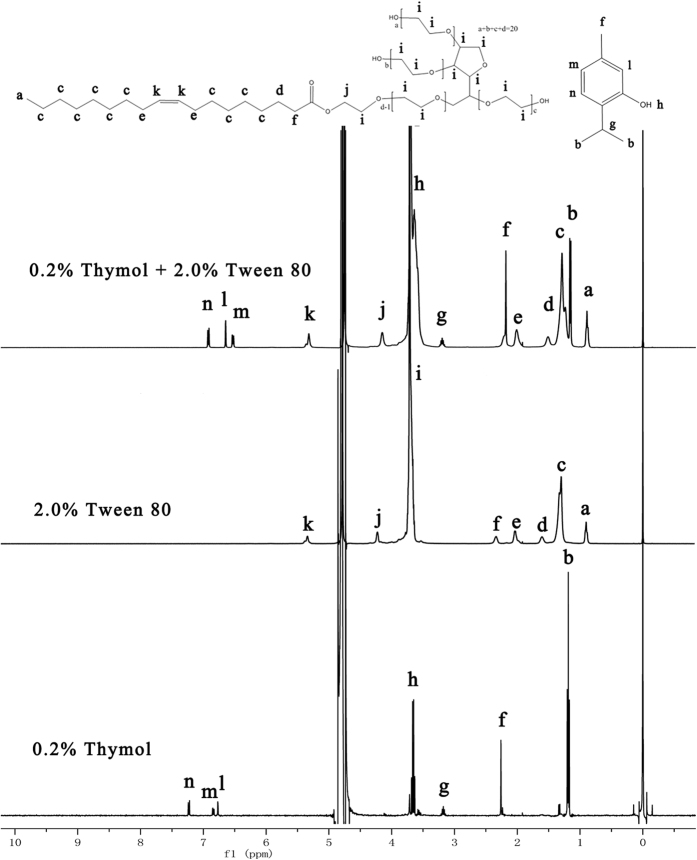
^1^H NMR spectra of a D_2_O solution of 0.2% thymol, 2.0% Tween 80 solution, and 0.2% thymol solubilized in 2.0% Tween 80 micelles.

^1^H NMR is a particularly useful tool to investigate the localization of molecules within surfactant micelles^25^. The ^1^H NMR spectra and peak assignments were shown in Fig. 3 and Table 1, respectively. Tween 80 exhibited eight kinds of protons in its spectra and only three of them shifted upfield significantly after solubilizing thymol in the micelles (Table 1). The shifted protons were located near the ester group between the hydrophilic head group and the oleic acid tail group, suggesting that thymol located near the ester group between the hydrophilic and hydrophobic regions of Tween 80 molecules. The ^1^H NMR spectra of thymol showed seven kinds of protons, three of which shifted upfield significantly after thymol solubilization. The shifted protons were all located on the benzene ring, and 3-H and 4-H shifted more significantly than 6-H, indicating that 3-H and 4-H side interacted with the ester group of Tween 80 by Van der Waals’ force. Solubilization is known to occur at a number of different sites in micelles: (1) at the micelle-water interface, (2) between the hydrophilic head groups, (3) in the palisade layer of the micelle, and (4) in the inner hydrophobic core of the micelle^26^. In this study, we propose that the thymol molecules are located at the junction of hydrophilic head groups and the palisade layer of Tween 80 micelles, allowing thymol to directly interact with the Tween 80 monomers.

### Radical scavenging activity

**Figure 4:**
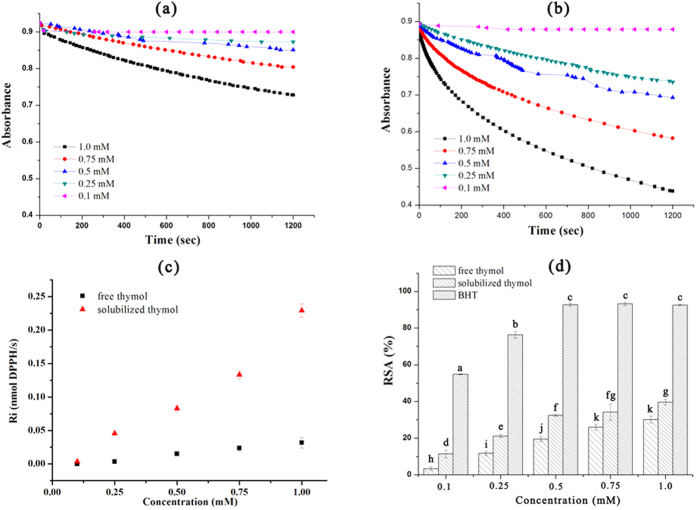
The visible absorbance change of DPPH after addition of (**a**) free and (**b**) solubilized thymol at various concentrations; (**c**) The initial reaction rate (R_i_) of free and solubilized thymol at various concentrations; (**d**) DPPH scavenging activity of free and solubilized thymol, and free BHT within 20 min.

**Figure 5:**
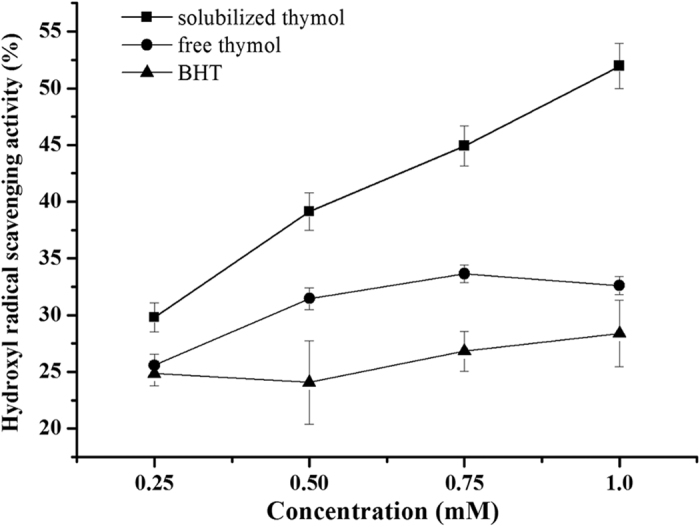
Hydroxyl radical scavenging activity of free and solubilized thymol, and free BHT after subtracting the solvent blank.

Dynamic absorbance curves for DPPH radical scavenging of the free and solubilized thymol were shown in Fig. 4a and b. During the determination time, the absorbance decreased with time and the radical scavenging activity increased with the increasing thymol concentration. At a very low concentration of 0.1 mM, both free and solubilized thymol similarly showed a very slow reaction with DPPH, as reflected by the same R_i_ value (Fig. 4c). With the increasing concentration, thymol in micelles showed a faster reaction with DPPH compared to the free thymol, and the ∆Ri value increased accordingly. The total DPPH RSA of free thymol, thymol in micelles, and free BHT within 20 min were shown in Fig. 4d. BHT showed an excellent scavenging activity even at 0.1 mM, and scavenged almost all radicals above the concentration of 0.5 mM. There are significant differences in DPPH RSA at each concentration between the free and solubilized thymol. The hydroxyl radical scavenging activities of free thymol, solubilized thymol, and BHT at various concentrations were shown in Fig. 5. The solubilized thymol showed a much higher hydroxyl radical scavenging activity than free thymol and BHT at all concentrations. However, BHT showed a weaker hydroxyl radical scavenging activity compared to free thymol, which is contrary to the results of DPPH radical scavenging activity.

The improved radical scavenging activity of solubilized antioxidants has also been observed by many other researchers. Hamed *et al*. found that incorporation of eugenol in Tween 20 micelles improved its DPPH radical scavenging activity^27^. β-carotene incorporated in chitosan-graft-poly (lactide) micelles showed a significantly higher DPPH radical scavenging activity than free β-carotene^24^. Noipa *et al*.^28^ observed a faster reaction between gallic acid and DPPH incorporated in Triton X-100, SDS, or CTAB micelles than those in methanol, which allowed shorter analysis time. The ability of the micellar curcumin to scavenge the ABTS radicals was higher than that of free curcumin^29^. A recent study demonstrated that the activity and rate of the DPPH RSA of naringenin increased with the increasing concentrations of SDS and CTAB micelles^30^. On the other hand, some contrary results have been also reported. Kim *et al*.^23^ found that the antioxidant activity of tea tree essential oil formulated in micelles was less than that of the crude essential oil. Chat *et al*.^15^ observed that the DPPH RSA of rutin in all micellar solutions became poorer compared to rutin in methanol.

The improved radical scavenging activity may be due to multiple factors. It was known that the close proximity of radicals to antioxidant is a crucial prerequisite for the radical reducing action of antioxidants^13^. It has been reported that the DPPH radical scavenging rate is enhanced in micellar solutions compared to that in methanol due to incorporation of DPPH inside the hydrophobic core of the micelle structure consequently leading to easier abstraction of phenol H-atom by DPPH^28^. Thymol is located between the hydrophilic head group and the hydrophobic tail group of Tween 80, which is actually the outer shell of the micelles. When micelles were added to DPPH methanol solution, DPPH molecules were easily extracted into the hydrocarbon core of micelles, leading to a much higher collision probability between thymol and DPPH^30^. Solvent is another important factor that affects the RSA value. Water is a known proton transfer medium that supports ionization of solutes^31^. Previous studies have shown that water could accelerate the DPPH radical scavenging reaction, which accounts partially for the increased reaction rate of DPPH radical scavenging by the solubilized thymol^32^.

### Reducing antioxidant activity

The TEAC coefficients of solubilized thymol in the Tween 80 solution, free thymol and BHT in methanol by the FRAP and CUPRAC assays were shown in Table 2. The results obtained from the CUPRAC assay were generally higher than the FRAP assay, in agreement with the results by Çelik *et al*. and Stef *et al*.^33^,^34^. CUPRAC assay was carried out at a nearly physiological pH (pH 7), while FRAP assay was conducted at an acidic condition (pH 3.6). At a more acidic condition, the reducing capacity may be suppressed due to protonation on antioxidants^35^. There is no difference in the reducing antioxidant activity between the free and solubilized thymol, but both are slightly poorer than BHT. Ge *et al*. found that β-carotene micelles showed a higher FRAP value than free carotene^24^. But the FRAP values were nearly the same after abstracting the micelle blank value. Hence, we assume here that the reducing power of antioxidants cannot be easily changed by changing the physical environment, based on the single electron transfer principle of FRAP and CUPRAC assays, which resemble the redox titration in classical chemical analysis^36^.

## Conclusions

We successfully improved the water solubility of thymol by solubilizing it in Tween 80 micelles. The results of NMR spectra indicated that the thymol molecules are located between the hydrophilic head group and the hydrophobic tail group of Tween 80. It can be concluded that the solubilized thymol not only maintained the reducing antioxidant activity, but also showed improved free radical scavenging activity in aqueous environments. Considering the undesired characteristics of hydrophobicitiy and specific aromas that may prevent the practical applications of thymol in food industry, the present study suggests a possibility of preparing thymol-carrying Tween 80 micelles with effective antioxidant activities, and provides a novel option enabling thymol-containing micelles to be applied in food products as the replacement of synthetic antioxidants, especially in clear beverages.

## Materials and Methods

### Materials

Thymol, Polyoxyethylene sorbitan mono-oleate (Tween80) (critical micelle concentration of Tween 80 is 0.002 wt%), 2,4,6-tri(2-pyridinyl)-1,3,5-triazine (TPTZ), and 2,2-diphenyl-1-picrylhydrazyl (DPPH), were purchased from Sigma Aldrich (St. Louis, MO, USA). Neocuproine (2,9-dimethyl-1, 10-phenanthroline), copper (II) chloride dihydrate, trichloroacetic acid (TCA), 2-thiobarbituric acid (TBA), and 2-deoxy-D-ribose were purchased from Aladdin-reagent Inc. (Shanghai, China). The other reagents were purchased from Sinopharm Chemical Reagent Co., Ltd., China. All reagents were used without further purification and used water was double-distilled.

### Maximum additive concentration

Based on our previous work, 2.0% (w/v) Tween 80 was chosen to form a micellar solution^37^,^38^. The maximum additive concentration was measured as described by Rao and McClements^39^. Briefly, aliquots of stock emulsion (10% thymol and 2.0% Tween 80) were added to 2.0% (w/v) Tween 80 solutions to give a range of final thymol concentrations (0.1% to 1.0%, w/v). The maximum solubilization was determined by measuring the absorbance of 2.0% Tween 80 solution added with different concentrations of thymol at 630 nm using a UV-visible spectrophotometer (UV-752, Shanghai Spectrum Instruments Co., Ltd, Shanghai, China).

### Characterization of micelles

The particle sizes of the empty micelles, thymol solubilized micelles, and free thymol in water were measured using the dynamic light scattering Nano-S90 (Malvern Instruments, UK). Measurements were conducted at 25 °C with a fixed angle of 90°.

TEM based on a method by Ho *et al*.^40^ was used to observe the morphology of micelles. The thymol solubilized Tween 80 micellar solution was dropped onto a 300 mesh carbon-coated cooper grid and negatively stained with 2% phosphotungstic acid, then they were dried in a desiccator for overnight and analyzed in a FEI Tecnai Spirit Biotwin G2 microscope (Hillsboro, OR, USA).

^1^H NMR spectra were recorded with a Bruker Avance 400 NMR spectrometer (Bruker, Germany) operating at 400 MHz using D_2_O as solvent. Spectra were acquired in regular one pulse sequences with a 30 degree proton pulse and a 3 s relaxation delay at room temperature.

### DPPH free radical scavenging assay

The DPPH free radical scavenging activities were measured as previously reported by Xie and Schaich^41^. 10 mM of solubilized thymol solution was prepared by dissolving 1.5 g of Tween 80 and 0.15 g of thymol in water and diluting to 100 mL. Free thymol and free BHT at a concentration of 10 mM were prepared in methanol. Sample solutions (200 μL) with various concentrations (10, 7.5, 5.0, 2.5, and 1.0 mM) were quickly added to 1.8 mL of freshly prepared 100 μM DPPH solution and mixed rapidly by aspiration. The absorbance change at 517 nm on a UV-Vis spectrophotometer (Shanghai Spectrum Instruments Co. Ltd., China) within 20 min were recorded by a digital camera, and then analyzed by Corel Video Studio to sketch the dynamic absorbance curve. The total DPPH radical scavenging activity within 20 min was measured in triplicate in dark. The blank was prepared as above by replacing the test sample with equivalent methanol. The radical scavenging activity (RSA%) was calculated as follow:


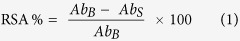


where, Ab_B_ and Ab_S_ are the absorbance values of the blank and sample, respectively. The measurements were carried out in triplicate.

Initial reaction rates (R_i_) were calculated from the initial absorbance drop (∆A_i_) within 60 s, determined by completion of reaction or regression equations with R^2^ > 0.9. ∆A_i_ was converted to ∆[DPPH]_i_, and the initial rate (R_i_) was recorded directly as nmols DPPH quenched/s (nmol DPPH/s).

### Hydroxyl radical scavenging assay

The deoxyribose degradation assay was used to evaluate the hydroxyl radical scavenging activity^42^. Using n-hexane as the solvent of free thymol and BHT to decrease the solvent interference to the greatest extent^43^, the measurements were conducted based on the method of Bektaşoğlu *et al*.^44^ with modifications. 1.5 mL of phosphate buffer (0.2 M, pH 7.4), 0.5 mL of 10 mM 2-deoxy-D-ribose (probe), 0.25 mL of 20 mM Na_2_EDTA, 0.25 mL of 20 mM FeSO_4_ solution, 2 mL of the sample solution with various concentrations (0.25, 0.50, 0.75, and 1.0 mM) or 2 mL of solvent without thymol, and 0.5 mL of 10 mM H_2_O_2_ were rapidly added to a tube in this order. The mixture was incubated for 30 min in a water bath kept at 50 °C. At the end of this period, the reaction was stopped by adding 2.5 mL of 2.8% TCA and 2.5 mL of 1% TBA, and the reaction mixture was kept in a 100 °C water bath for 15 min. The test tubes were cooled under a flow of running tap water, then an equivalent amount of water was added to compensate the vaporized n-hexanol, and the absorbance at 520 nm on a UV-Vis spectrophotometer (Shanghai Spectrum Instruments Co. Ltd., China) were recorded.

The hydroxyl radical scavenging activity expressed as:


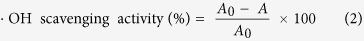


where A_0_ and A are the TBARS absorbance of the system in the absence and presence of scavenger, respectively.

### FRAP assay

Ferric reducing activity of thymol was measured as described by Benzie and Strain^45^. The FRAP reagents was prepared freshly by mixing 10 mM TPTZ, 20 mM FeCl_3_ and 300 mM acetate buffer (pH 3.60) in a ratio of 1:1:10 (v/v/v). Sample solutions (5 μL) were added into 180 μL of FRAP reagents in a 96-well microplate. The mixtures were shaken, incubated at 37 °C in dark for 30 min and then A593 nm readings were recorded using a Tecan Infinite M200 Pro instrument (Mannedorf, Switzerland). The standard curve was established using FeSO_4_, and the results were expressed as TEAC (Trolox equivalent antioxidant capacity). All measurements were performed in triplicate.

### CUPRAC assay

The CUPRAC measurements were conducted based on the method of Apak *et al*.^46^ with modifications. Copper (II) chloride solution at a concentration of 10^−2^ M, ammonium acetate (NH_4_Ac) buffer at pH 7.0 at a concentration of 1 M were prepared by dissolving it in water. The neocuproine (Nc) solution at a concentration of 7.5 × 10^−3^ M was prepared by dissolving 0.039 g of Nc in 95% EtOH and diluting to 25 mL with ethanol. 1 mL of Copper (II) chloride solution, 1 mL of Nc solution, 1 mL of NH_4_Ac buffer, 1 mL of the antioxidant solution, and 1 mL of H_2_O for free thymol and BHT or 1 mL of methanol for the solubilized thymol were rapidly added to a tube in this order.

Water or methanol was added to eliminate the solvent effect. The tubes were stoppered, and after 1 h in a 37 °C water bath the absorbance at 450 nm were recorded against a reagent blank. The results were expressed as TEAC (Trolox equivalent antioxidant capacity). All measurements were performed in triplicate.

### Statistical analysis

Results are presented as mean ± standard deviation (SD) and analyzed by SPSS software (version 16.0). Statistical significance of mean values between multiple treatment groups was accessed by one-way analysis of variance (ANOVA) with Turkey’s test. *P* value < 0.05 was considered statistically significant.

## Additional Information

**How to cite this article**: Deng, L.-L. *et al*. Physical characterization and antioxidant activity of thymol solubilized Tween 80 micelles. *Sci. Rep.* **6**, 38160; doi: 10.1038/srep38160 (2016).

**Publisher's note:** Springer Nature remains neutral with regard to jurisdictional claims in published maps and institutional affiliations.

## Figures and Tables

**Table 1:** ^1^H-NMR chemical shifts of different functional groups of thymol, Tween 80 before and after solubilization, respectively.

Thymol (ppm)				Tween 80 (ppm)			
Functional group	Before (δ^1^)	After (δ^2^)	∆δ (δ^2^–δ^1^)	Functional group	Before (δ^3^)	After (δ^4^)	∆δ (δ^4^–δ^3^)
**CH**_**3**_	1.18	1.15	−0.03	**CH**_**3**_	0.9	0.88	−0.02
**CH**_**3**_	2.26	2.18	−0.08	**CH**_**2**_	1.3	1.29	−0.01
**CH**	3.15	3.2	0.05	**CH**_**2**_**CH**_**2**_**OCO**	1.6	1.51	−0.09
**OH**	3.63	3.63	0	**CH**_**2**_**CH=CHCH**_**2**_	2.03	2.01	−0.02
**6-H**	6.75	6.63	−0.12	**CH**_**2**_**OCO**	2.33	2.18	−0.15
**4-H**	6.83	6.52	−0.31	**CH**_**2**_	3.71	3.72	0.01
**3-H**	7.22	6.91	−0.31	**OCOCH**_**2**_	4.25	4.15	−0.1
				**CH = CH**	5.34	5.32	0

**Table 2:** Trolox equivalent antioxidant capacities of free and solubilized thymol, and free BHT calculated with respect to the FRAP and CUPRAC methods.

	TEAC_FRAP_	TEAC_CUPRAC_
Free thymol	0.74 ± 0.05^a^	0.95 ± 0.02^c^
Solubilized thymol	0.79 ± 0.07^a^	0.96 ± 0.02^c^
BHT	0.85 ± 0.07^b^	1.04 ± 0.03^d^
